# Exposure to *Mycobacterium tuberculosis* during Flexible Bronchoscopy in Patients with Unexpected Pulmonary Tuberculosis

**DOI:** 10.1371/journal.pone.0156385

**Published:** 2016-05-26

**Authors:** Hae Jung Na, Jung Seop Eom, Geewon Lee, Jeong Ha Mok, Mi Hyun Kim, Kwangha Lee, Ki Uk Kim, Min Ki Lee

**Affiliations:** 1 Department of Internal Medicine, Pusan National University School of Medicine, Busan, Korea; 2 Department of Radiology, Pusan National University School of Medicine, Busan, Korea; 3 Biomedical Research Institute, Pusan National University Hospital, Busan, Korea; Hospital San Agustín. Aviles. Asturias. Spain, SPAIN

## Abstract

**Objective:**

Recent guidelines recommend the use by healthcare personnel of a fit-tested N95 particulate respirator or higher-grade respiratory precaution in a patient undergoing bronchoscopy when pulmonary tuberculosis (PTB) is suspected. However, PTB may be unexpectedly diagnosed in this setting and therefore not evaluated, resulting in the unexpected exposure to *Mycobacterium tuberculosis* (MTB) of healthcare workers in the bronchoscopy suite. Here, we examined the incidence of unexpected exposure to MTB during flexible bronchoscopy and determined the exposure-related factors.

**Methods:**

Between 2011 and 2013, a retrospective study was conducted to evaluate unexpected diagnoses of PTB in the bronchoscopy suite. During the study period, 1650 consecutive patients for whom previous CT scans were available and who underwent bronchoscopy for respiratory disease other than PTB were included. The results of bronchial washing, bronchoalveolar lavage, and post-bronchoscopic sputum were reviewed.

**Results:**

PTB was unexpectedly diagnosed in 76 patients (4.6%). The presence of anthracofibrosis [odds ratio (OR), 3.878; 95% confidence interval (CI), 1.291–11.650; *P* = 0.016), bronchiectasis (OR, 1.974; 95% CI, 1.095–3.557; *P* = 0.024), or atelectasis (OR, 1.740; 95% CI, 1.010–2.903; *P* = 0.046) as seen on chest CT scan was independently associated with unexpected PTB. Patients with both anthracofibrosis and atelectasis were at much higher risk of unexpected PTB (OR, 4.606; 95% CI, 1.383–15.342; *P* = 0.013).

**Conclusions:**

The risk of MTB exposure by healthcare personnel in the bronchoscopy suite due to patients with undiagnosed PTB has been underestimated. Therefore, in geographic regions with an intermediate PTB prevalence, such as South Korea (97/100,000 persons per year), higher-grade respiratory precaution, such as a fit-tested N95 particulate respirator, should be considered to prevent occupational exposure to MTB during routine bronchoscopy, especially in patients with CT-confirmed anthracofibrosis, bronchiectasis, or atelectasis.

## Introduction

Flexible bronchoscopy is a valuable diagnostic method for various neoplastic and non-neoplastic lung diseases [1,2]. In patients with suspected pulmonary tuberculosis (PTB) in whom the sputum smear is negative for acid-fast staining, bronchoscopy with bronchial washing or bronchoalveolar lavage (BAL) is particularly useful to verify the diagnosis [3,4]. During bronchoscopy of these patients, healthcare workers must take the necessary precautions to minimize their risk of exposure to *Mycobacterium tuberculosis* (MTB). According to the American College of Chest Physicians and American Association for Bronchology Consensus Statement, during bronchoscopy of patients with suspected PTB, the bronchoscopist should wear a fit-tested N95 particulate respirator to minimize the risk of exposure to airborne MTB [5].

Besides patients with clinically or radiologically suspected PTB, those whose respiratory pathologies are initially not thought to be PTB are indeed occasionally diagnosed with the disease based on routine culture or polymerase chain reaction (PCR) for MTB using bronchial washing fluid or BAL [6,7]. The incidence of PTB and thus of the unexpected exposure of healthcare personnel to MTB is likely to be higher in areas with an intermediate or high prevalence of TB than in countries where TB has a low prevalence, such as the United States. However, unexpected exposure to MTB has not been examined nor is it addressed by the above-mentioned guidelines.

The aim of this retrospective study was to determine the incidence of unexpectedly diagnosed PTB using the bronchoscopy specimens (bronchial washing fluid and BAL) and post-bronchoscopy sputum of patients not initially diagnosed with the disease. The results were interpreted as an estimate of the unexpected exposure to MTB by healthcare workers in the bronchoscopy suite. We also sought to identify the independent factors related to unexpectedly diagnosed PTB.

## Methods and Materials

### Study population

**Fig 1:**
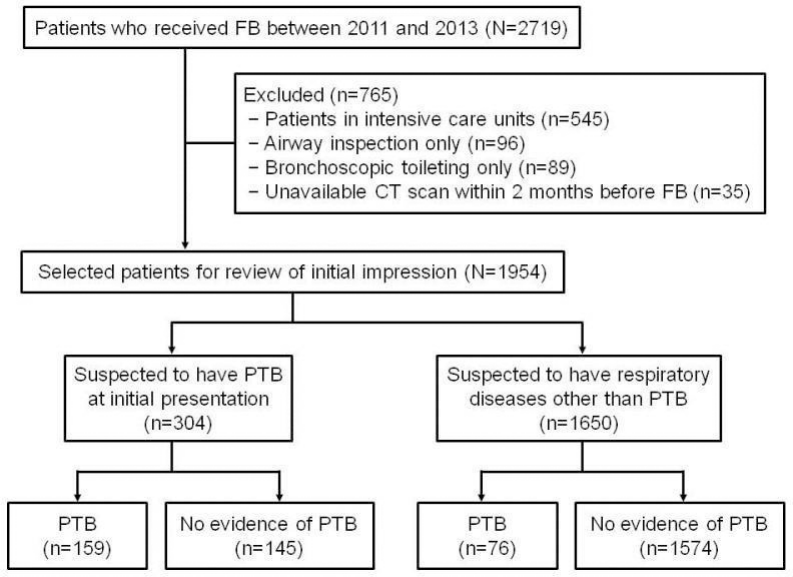
Study flow diagram. FB, flexible bronchoscopy; PTB, pulmonary tuberculosis.

This retrospective study was based on 2719 consecutive patients who underwent flexible bronchoscopy between January 2011 and December 2013 at Pusan National University Hospital, Busan, South Korea. This region of the country has an intermediate incidence of PTB (97/100,000 person per year) [8]. Of these patients, 765 were excluded for the following reasons: 1) hospitalization in the intensive care unit (n = 545); 2) bronchoscopy performed for airway inspection only (n = 96); 3) bronchoscopic toileting performed without bronchial washing or BAL (n = 89); and 4) chest CT scans had not been performed within 2 months before bronchoscopy (n = 35). Thus, the electronic medical records of 1954 patients were reviewed to identify the initial diagnostic impression recorded by the pulmonary physician. A “suspicion of PTB” was defined as PTB suspected by a pulmonary physician on review of clinical and radiological data. Other patients were considered to be under suspicion of respiratory diseases other than PTB. Finally, 1650 patients whose presumptive diagnosis was respiratory disease other than PTB were selected to determine the incidence of unexpected exposure to MTB in healthcare workers in the bronchoscopy suite (Fig 1).

The Institutional Review Board of Pusan National University Hospital approved this study, including the review and publication of information obtained from the patients’ records. The need for informed consent was waived, due to the retrospective nature of the study (IRB no. E-2015059). All patients information were anonymized preceding the analysis.

### Bronchoscopic procedure and post-bronchoscopy sputum

After conscious sedation with midazolam and fentanyl, the patients underwent bronchoscopy transorally or transnasally. The examination was performed by board-certified respiratory physicians using a flexible fiber-optic bronchoscope (Olympus, Tokyo, Japan). Before the bronchoscopy, oropharyngeal and laryngeal anesthesia was obtained by the administration of 2 mL of nebulized 4% lidocaine, followed by 1 mL of 2% topical lidocaine sprayed into the patient’s oral and nasal cavities. After the bronchoscope was advanced through the vocal cords, 2 mL of 2% lidocaine solution was instilled into the trachea and both main bronchi through the working channel of the bronchoscope. If needed, additional 2% lidocaine solutions were infused during the procedure.

After the initial exploration of the tracheobronchial tree by the bronchoscopist, bronchial washing fluid was obtained in the trap bottle by the instillation of 10 mL of sterile normal saline into the affected segmental bronchus, followed by immediate suction. The procedure was repeated two or three times until 10 mL of material was retrieved for microbiological and cytological examination. BAL was performed using standard techniques as previously described [9]. Collected bronchial washing or BAL fluids were analyzed by fluorescence microscopy using auramine-rhodamine staining. Mycobacteria were cultured in liquid culture medium (BacT/ALERT MP; bioMérieux, Durham, NC) and in 3% Ogawa medium. Real-time PCR for MTB was performed using the AdvanSure TB/NTM real-time PCR kit (LG Life Science, Seoul, South Korea).

Post-bronchoscopy sputum was collected immediately after bronchoscopy as described in a previous study [10] and used for the staining, culture, and PCR analysis of MTB.

### Diagnosis of PTB

The presence of MTB in cultured bronchoscopy samples confirmed a diagnosis of PTB (bacteriologically confirmed PTB). PTB was also diagnosed in patients whose real-time PCR was positive for MTB and who showed clinical and radiological improvement after standard anti-TB treatment [9].

### Analysis of the CT scans

All chest CT scans were reviewed by one radiologist who was blinded to the final diagnosis and to the results of MTB staining, culture, and PCR analysis. The CT-based diagnoses were as follows: 1) pneumonia, 2) bronchiolitis, 3) lung nodule, 4) lung mass, 5) anthracofibrosis of the airway, 6) bronchiectasis, 7) atelectasis, 8) interstitial lung disease, 9) airway stenosis, 10) pleural effusion, and 11) fibrocalcific parenchymal PTB. The CT scan criteria used have been described in previous studies. For example, bronchiectasis was considered present based on the visibility of a bronchus in the outer one-third of the lung or an inner lumen diameter exceeding that of the accompanying pulmonary artery [10]. Atelectasis was considered present when there was increased attenuation of the affected lung associated with a reduced lung volume [11]. Anthracofibrosis of the airways was defined as bronchial narrowing or obstruction and/or bronchial wall thickening [12]. The others are listed in a table in S1 Table.

### Statistical analysis

All results are presented as numbers (percentages) or medians (interquartile range, IQR), as appropriate. The data were compared using the χ2 or Fisher’s exact test for categorical variables and the Mann–Whitney U-test for continuous variables. Logistic regression analyses were performed to re-examine the factors with a significance of *P* < 0.1 in the univariate analyses. *P*-values < 0.05 were considered to indicate statistical significance. SPSS for Windows version 17.0 (SPSS Inc., Chicago, IL) was used for the statistical analyses.

## Results

### Study population

Of the initial 1,954 patients, 304 with presumptive diagnoses of PTB were excluded and 159 (52.3%) were ultimately diagnosed with PTB (Fig 1). Finally, 1,650 patients whose presumptive diagnoses were respiratory diseases other than PTB were studied to determine the incidence of unexpected exposure of healthcare workers to MTB in the bronchoscopy suite. The characteristics of patients with suspected PTB and those suspected of having respiratory diseases other than PTB are shown in Table 1.

**Table 1:** Data on patients with suspected PTB and respiratory diseases other than PTB.

Variable	Suspected to have PTB (n = 304)	Suspected to have respiratory diseases other than PTB (n = 1650)	*P*-value
Age, years	57 (44–68)	65 (57–72)	< 0.001
Male gender	182 (59.9)	1040 (63.0)	0.295
Body mass index, kg/m^2^	21.6 (20.0–23.7)	22.9 (20.9–24.5)	< 0.001
Current or ex-smoker	124 (40.8)	789 (47.8)	0.024
Comorbidities, overlapping			
Malignancy*	48 (15.8)	746 (45.2)	< 0.001
Cardiovascular disease	62 (20.4)	524 (31.8)	< 0.001
Diabetes mellitus	44 (14.5)	236 (14.3)	0.921
Chronic kidney disease	7 (2.3)	32 (1.9)	0.671
Hematologic disease	2 (0.7)	12 (0.7)	0.627
Liver disease	8 (2.6)	45 (2.7)	0.932
Rheumatologic disease	5 (1.6)	21 (1.3)	0.598
Chest CT findings, overlapping			
Pneumonia	95 (31.3)	487 (29.5)	0.622
Bronchiolitis	171 (56.3)	152 (9.2)	< 0.001
Lung nodule	175 (57.6)	722 (43.8)	< 0.001
Lung mass	30 (9.9)	556 (33.7)	< 0.001
Anthracofibrosis	0 (0)	23 (1.4)	0.020
Bronchiectasis	50 (16.4)	221 (13.4)	0.157
Atelectasis	35 (11.5)	289 (17.5)	0.010
Interstitial lung disease	4 (1.3)	131 (7.9)	< 0.001
Airway stenosis	17 (5.6)	66(4.0)	0.206
Pleural effusion	58 (19.1)	245 (14.8)	0.061
Parenchymal calcification	49 (16.1)	171 (10.4)	0.004

PTB, pulmonary tuberculosis.

* Lung cancer was detected in 12 (25%) and 646 (86.6%) of the patients with malignancy in the group of suspected to have PTB and respiratory diseases other than TB, respectively.

In 1,650 patients whose presumptive diagnoses were respiratory diseases other than PTB, 76 (4.6%) were unexpectedly diagnosed with PTB; their baseline characteristics are summarized in Table 2.

**Table 2:** Baseline characteristics of 76 patients who unexpectedly diagnosed with PTB.

Variable	No. (%) or median (interquartile range)
Age, years	65 (58–73)
Male gender	34 (44.7)
Height, cm	160.0 (153.0–169.0)
Weight, kg	57.5 (50.8–64.8)
Body mass index, kg/m^2^	22.4 (20.6–24.9)
Smoking history	
Never smoked	49 (64.5)
Current or ex-smoker	27 (35.5)
Comorbidities, overlapping	
Malignancy	24 (31.6)
Cardiovascular disease	20 (26.3)
Diabetes mellitus	9 (11.8)
Chronic kidney disease	1 (1.3)

PTB, pulmonary tuberculosis.

### Bronchoscopy specimens

Of the 1650 patients whose presumptive diagnosis was respiratory disease other than PTB, bronchial washing and BAL fluids were obtained from 1514 (91.8%) and 136 (8.2%), respectively. Post-bronchoscopy sputum was collected in 1425 of the 1650 patients (86.4%). The MTB culture and PCR results are presented in Table 3. PTB was diagnosed in 49 patients (3.0%) based on the MTB culture. PCRs using any bronchoscopy specimen were MTB-positive in 42 patients (2.5%). An additional 27 patients (1.6%) without bacteriological confirmation were diagnosed with PTB based on the PCR results and radiological and clinical improvement after standard anti-TB treatment. Thus, 76 of the 1650 patients (4.6%) were eventually diagnosed with PTB.

**Table 3:** Results of MTB culture and PCR.

	MTB-positive culture (%)	MTB-positive PCR (%)
Bronchial washing*	31/1514 (2.0)	32/1514 (2.1)
Bronchoalveolar lavage	2/136 (1.5)	2/136 (1.5)
Post-bronchoscopy sputum^†^	36/1425 (2.5)	13/310 (4.2)^‡^
All bronchoscopy specimens^§^	49/1650 (3.0)	42/1650 (2.5)

MTB, *Mycobacterium tuberculosis*; PCR, polymerase chain reaction.

*Fifteen patients had both MTB-positive cultures and MTB-positive PCR.

^†^Five patients had both MTB-positive cultures and MTB-positive PCR.

^‡^PCR for MTB was performed in 310 of the 1425 patient with post-bronchoscopy sputum specimens.

^§^Fifteen patients had both MTB-positive cultures and MTB-positive PCR.

### Factors contributing to an unexpected diagnosis of PTB

The characteristics of the patients with and without a diagnosis of PTB are compared in Table 4. Among the patients with PTB, fewer were male (*P* = 0.001) and had a smoking history (*P* = 0.028). Significantly fewer patients had an underlying malignancy, including lung cancer, in the group with PTB compared with the group without PTB (*P* = 0.014), while other comorbidities did not differ significantly. There was also no significant difference between the groups in terms of age or body mass index. However, patients with PTB were significantly more likely than those without PTB to present with bronchiolitis (18.4 vs. 8.8%, *P* = 0.004), anthracofibrosis (7.9 vs. 1.1%, *P* < 0.001), bronchiectasis (26.3 vs. 12.8%, *P* = 0.001), atelectasis (32.9 vs. 16.8%, *P* < 0.001), or airway narrowing (9.2 vs. 3.7%, *P* = 0.029) as determined on chest CT. Conversely, lung mass seen on chest CT was higher in the patients without than with PTB (34.2 vs. 22.4%, *P* = 0.032).

**Table 4:** Data of the patients with and without PTB.

	With PTB (n = 76)	Without PTB (n = 1574)	*P*-value
Age, years	65 (58–73)	65 (57–72)	0.507
Male	34 (44.7)	1006 (63.9)	0.001
Body mass index, kg/m^2^	22 (21–25)	23 (21–25)	0.724
Current or ex-smoker	27 (35.5)	762 (48.4)	0.028
Comorbidities, overlapping			
Diabetes	9 (11.8)	227 (14.4)	0.530
Malignancy*	24 (31.6)	722 (45.9)	0.014
Cardiovascular disease	20 (26.3)	504 (32.0)	0.297
Chronic kidney disease	1 (1.3)	31 (2.0)	0.686
Hematologic disease	0 (0.0)	12 (0.8)	0.445
Liver disease	0 (0.0)	45 (2.9)	0.266
Rheumatologic disease	0 (0.0)	21 (1.3)	0.621
Chest CT finding, overlapping			
Pneumonia	26 (34.2)	461 (29.3)	0.358
Bronchiolitis	14 (18.4)	138 (8.8)	0.004
Lung nodule	31 (40.8)	691 (43.9)	0.593
Lung mass	17 (22.4)	539 (34.2)	0.032
Anthracofibrosis	6 (7.9)	17 (1.1)	< 0.001
Bronchiectasis	20 (26.3)	201 (12.8)	0.001
Atelectasis	25 (32.9)	264 (16.8)	< 0.001
Interstitial lung disease	4 (5.3)	127 (8.1)	0.377
Airway stenosis	7 (9.2)	59 (3.7)	0.029
Pleural effusion	10 (13.2)	235 (14.9)	0.671
Fibrocalcific parenchymal PTB	12 (15.8)	159 (10.1)	0.112

PTB, pulmonary tuberculosis.

* Lung cancer was detected in 18 (75%) and 628 (87%) of the patients with malignancy in the group with and without PTB, respectively.

Binary logistic regression analysis was performed to identify the independent risk factors associated with an unexpected diagnosis of PTB in patients undergoing bronchoscopy (Table 5). The presence of anthracofibrosis [odds ratio (OR), 3.878; 95% confidence interval (CI), 1.291–11.650; *P* = 0.016], bronchiectasis (OR, 1.974; 95% CI, 1.095–3.557; *P* = 0.024) or atelectasis (OR, 1.740; 95% CI, 1.010–2.903; *P* = 0.046) diagnosed on the basis of chest CT was independently associated with an unexpected diagnosis of PTB.

**Table 5:** Logistic regression analysis to identify factors associated with PTB.

	Odds ratio (95% confidence interval)	*P*-value
Age	1.010 (0.989–1.032)	0.338
Male	1.559 (0.850–2.861)	0.151
Body mass index, kg/m^2^	1.000 (0.999–1.001)	0.595
Smoking status	0.964 (0.522–1.782)	0.907
Malignant disease	0.777 (0.429–1.406)	0.404
Bronchiolitis	1.680 (0.870–3.244)	0.122
Lung mass	0.860 (0.444–1.666)	0.656
Anthracofibrosis	3.878 (1.291–11.650)	0.016
Bronchiectasis	1.974 (1.095–3.557)	0.024
Atelectasis	1.740 (1.010–2.903)	0.046
Airway stenosis	1.580 (0.640–3.903)	0.321

PTB, pulmonary tuberculosis.

### Subgroup analysis

Based on the results of the logistic regression analysis, we further analyzed the data to determine whether a combination of chest CT findings resulted in an even higher risk of an unexpected diagnosis of PTB. The presence of both anthracofibrosis and atelectasis, detected in 15 (0.9%) of our patients, independently increased the risk of an unexpected diagnosis of PTB (OR, 4.606; 95% CI, 1.383–15.342; *P* = 0.013), whereas the combination of bronchiectasis and atelectasis, detected in 47 patients (2.8%), did not (OR, 1.900; 95% CI, 0.746–4.840; *P* = 0.179). None of the chest CT scans of the patients demonstrated combined anthracofibrosis and bronchiectasis such that the effect of these two diseases could not be evaluated.

## Discussion

In this retrospective study, the incidence of unexpected diagnosis of PTB in the bronchoscopy suite was 4.6%. The independent risk factors associated with an unexpected diagnosis of PTB were anthracofibrosis, bronchiectasis, and atelectasis, diagnosed based on the chest CT findings. Patients with a combination of CT-confirmed anthracofibrosis and atelectasis were at much higher risk of an unexpected diagnosis of PTB. This is the first report regarding the risk of unexpected diagnosis of PTB in patients undergoing bronchoscopy and the related risk factors in a country with an intermediate TB burden. It thus demonstrates the incidence of unexpected occupational MTB exposure by healthcare workers in the bronchoscopy suite.

Several previous studies reported the usefulness of routine culture for MTB using bronchial washing fluid. In their study of a group of patients in the US, Kvale *et al*. reported a low incidence of PTB diagnosed from bronchial washings (3/859 patients, 0.3%) [13]. On the other hand, in India, a high-prevalence area, Sarkar *et al*. found that 9.1% of patients (15/164) had PTB, which was diagnosed using routine MTB culture of bronchial washing fluid. Overall, the proportion of patients in the bronchoscopy suite who had MTB-positive cultures was 0.3–1.3% in countries with a low PTB prevalence and 3.7–9.1% in countries with an intermediate or high PTB prevalence [14]. However, these studies were based on patients with suspected and unsuspected TB, such that they cannot be used to deduce the incidence of unexpected exposure to MTB.

Our results showed that patients with bronchiectasis, as seen on chest CT scan, have a significantly increased risk of an unexpected diagnosis of PTB during bronchoscopy (OR = 1.974). The most common etiologies of bronchiectasis include postinfectious conditions (bacteria, *Aspergillus* species, mycobacteria, viruses), genetic diseases, and immune deficiency [15]. Although MTB infection can induce traction bronchiectasis, the current guidelines do not recommend higher-grade respiratory precaution during bronchoscopy [5]. However, in areas with a relatively high incidence of TB, bronchiectasis may be more often associated with the disease than is the case in regions where the incidence is low [16,17]. Also, our study patients who had anthracofibrosis (OR, 3.878) on chest CT scans were at higher risk of an unexpected diagnosis of PTB. In fact, PTB is one of the most common diseases associated with bronchial anthracofibrosis [18]; in endobronchial TB without lung parenchymal lesions it can be difficult to distinguish between TB and anthracofibrosis. Indeed, on chest CT, anthracofibrosis could instead be or coexist with endobronchial TB [19]. Atelectasis on chest CT scans also increased the risk of an unexpected diagnosis of PTB during bronchoscopy (OR = 1.740). Atelectasis itself is included in the CT findings of anthracofibrosis [19]. In patients with subtle endobronchial TB not seen on CT, only distal atelectasis will be detectable. In addition, the presence of atelectasis will mask findings suggestive of PTB (such as a cavity or the tree-in-bud sign) [20].

PTB may be associated with atelectasis, bronchiectasis, and anthracofibrosis. Although none of these conditions are specific for PTB, they may coexist with it [21]. Therefore, when PTB is suspected in a low prevalence area of the disease, recent guidelines recommend the use of a fit-tested N95 particulate respirator during bronchoscopy examination of these patients. However, our results indicate that in intermediate-to-high prevalence areas, high-grade respiratory precaution should be considered when bronchoscopy is performed in patients with bronchiectasis, anthracofibrosis, or atelectasis as these patients may have undiagnosed PTB.

Several studies have examined the association between PTB and lung cancer. The association between PTB and lung cancer is controversial. Wu *et al*. found that lung cancer patients have a higher incidence of PTB [22], while Denholm *et al*. reported that there was no association between tuberculosis and lung cancer [23]. We analyzed the difference between the proportion of unexpected tuberculosis in patients with and without lung cancer. Interestingly, the incidence of unexpected PTB was higher in the group without lung cancer than in the lung cancer group (5.8% vs. 2.8%). We believe that further large-scaled prospective studies are needed to verify the association between pulmonary tuberculosis and lung cancer.

The limitations of this study included a retrospective design and exclusion of patients who did not undergo CT prior to bronchoscopy, such that selection bias may have influenced our results. A prospective study would be needed to fully validate our findings. Second, our study was conducted at a single institution in an area of intermediate PTB burden, which may limit the application of our findings in areas of differing PTB prevalence. Third, we diagnosed PTB in patients with MTB-negative cultures but MTB-positive PCR findings when clinical and radiological improvements were evident after standard anti-tuberculosis treatment. As our work was retrospective in nature, potential misdiagnoses (false-positive PCR data) could not be excluded. Fourth, analysis by two or more radiologists, who are in good agreement, is generally required when radiological data are evaluated. Here, however, one radiologist reviewed all chest CT scans. Fifth, post-bronchoscopy sputum was not collected from all patients. Finally, whether patients with an unexpected diagnosis of PTB actually expose healthcare workers in the bronchoscopy suite to MTB is not clear. However, because flexible bronchoscopy is a cough-inducing procedure, the probability of MTB transmission to healthcare workers at bronchoscopy by patients with undetected PTB cannot be ruled out.

In conclusion, the incidence of an unexpected diagnosis of PTB as determined from bronchoscopic specimens or post-bronchoscopy sputum was relatively high, which points out the underestimation of possible exposure to MTB during bronchoscopy. Our study also demonstrated that patients with CT-confirmed anthracofibrosis, bronchiectasis, or atelectasis have a higher risk of PTB, which, if left undiagnosed, may lead to the exposure of healthcare workers in the bronchoscopy suite to MTB. Thus, in regions with intermediate TB prevalence, we suggest the use during routine bronchoscopy of a fit-tested N95 particulate respirator or higher-grade respiratory precaution to prevent occupational exposure to MTB in patients with bronchiectasis, atelectasis, or anthracofibrosis evident on CT.

## Supporting Information

S1 Table CT scan criteria. PTB, pulmonary tuberculosis.
(DOCX)

## References

[1] British Thoracic Society Bronchoscopy Guidelines Committee, a Subcommittee of Standards of Care Committee of British Thoracic Society. British Thoracic Society guidelines on diagnostic flexible bronchoscopy. Thorax. 2001;56(Suppl 1):i1–21. 11158709 10.1136/thorax.56.suppl_1.i1 PMC1765978

[2] Slade MG, Rahman NM, Stanton AE, Curry L, Slade GC, Clelland CA, et al Improving standards in flexible bronchoscopy for lung cancer. Eur Respir J. 2011;37:895–901. 10.1183/09031936.00097110 20693252

[3] Yoo H, Song JU, Koh WJ, Jeon K, Um SW, Suh GY, et al Additional role of second washing specimen obtained during single bronchoscopy session in diagnosis of pulmonary tuberculosis. BMC Infect Dis. 2013;13:404 10.1186/1471-2334-13-404 24059248 PMC3765986

[4] Schoch OD, Rieder P, Tueller C, Altpeter E, Zellweger JP, Rieder HL, et al Diagnostic yield of sputum, induced sputum, and bronchoscopy after radiologic tuberculosis screening. Am J Respir Crit Care Med. 2007;175:80–6. 17053204 10.1164/rccm.200608-1092OC

[5] Mehta AC, Prakash UB, Garland R, Haponik E, Moses L, Schaffner W, et al American College of Chest Physicians and American Association for Bronchology [corrected] consensus statement: prevention of flexible bronchoscopy-associated infection. Chest. 2005;128:1742–55. 16162783 10.1378/chest.128.3.1742 PMC7094662

[6] Bates M, O'Grady J, Mwaba P, Chilukutu L, Mzyece J, Cheelo B, et al Evaluation of the burden of unsuspected pulmonary tuberculosis and co-morbidity with non-communicable diseases in sputum producing adult inpatients. PLoS One. 2012;7:e40774 10.1371/journal.pone.0040774 22848401 PMC3407179

[7] Kim MH, Suh GY, Chung MP, Kim H, Kwon OJ, Lee JH, et al The value of routinely culturing for tuberculosis during bronchoscopies in an intermediate tuberculosis-burden country. Yonsei Med J. 2007;48:969–72. 18159588 10.3349/ymj.2007.48.6.969 PMC2628186

[8] World Health Organisation. 5 November 2014. Tuberculosis country profiles. Available: http://www.who.int/tb/country/data/profiles/en

[9] George PM, Mehta M, Dhariwal J, Singanayagam A, Raphael CE, Salmasi M, et al Post-bronchoscopy sputum: improving the diagnostic yield in smear negative pulmonary TB. Respir Med. 2011; 105:1726–31. 10.1016/j.rmed.2011.07.014 21840695

[10] Eom JS, Lee G, Lee HY, Oh JY, Woo SY, Jeon K, et al The relationships between tracheal index and lung volume parameters in mild-to-moderate COPD. Eur J Radiol. 2013;82:e867–72. 10.1016/j.ejrad.2013.08.028 24035456

[11] Hansell DM, Bankier AA, MacMahon H, McLoud TC, Müller NL, Remy J. Radiology. 2008;246:697–722. 10.1148/radiol.2462070712 18195376

[12] Kala J, Sahay S, Shah A. Bronchial anthracofibrosis and tuberculosis presenting as a middle lobe syndrome. Prim Care Respir J. 2008;17:51–5. 10.3132/pcrj.2008.00003 18253679 PMC6619869

[13] Kvale PA, Johnson MC, Wroblewski DA. Diagnosis of tuberculosis: routine cultures of bronchial washings are not indicated. Chest. 1979;76:140–2. 110539 10.1378/chest.76.2.140

[14] Laub RR, Sivapalan P, Wilcke T, Svensson E, Clementsen PF. Routine examination for tuberculosis is still indicated during bronchoscopy for pulmonary infiltrates. Dan Med J. 2015;62:pii:A5063 26050827

[15] Barker AF. Bronchiectasis. N Engl J Med. 2002;346:1383–93. 11986413 10.1056/NEJMra012519

[16] Byrne AL, Marais BJ, Mitnick CD, Lecca L, Marks GB. Tuberculosis and chronic respiratory disease: a systematic review. Int J Infect Dis. 2015;32:138–46. 10.1016/j.ijid.2014.12.016 25809770

[17] Kwak HJ, Moon JY, Choi YW, Kim TH, Sohn JW, Yoon HJ, et al High prevalence of bronchiectasis in adults: analysis of CT findings in a health screening program. Tohoku J Exp Med. 2010;222:237–42. 21127394 10.1620/tjem.222.237

[18] Kim HJ, Kim SD, Shin DW, Bae SH, Kim AL, Kim JN, et al Relationship between bronchial anthracofibrosis and endobronchial tuberculosis. Korean J Intern Med. 2013;28:330–8. 10.3904/kjim.2013.28.3.330 23682227 PMC3654131

[19] Kim HY, Im JG, Goo JM, Kim JY, Han SK, Lee JK, et al Bronchial anthracofibrosis (inflammatory bronchial stenosis with anthracotic pigmentation): CT findings. AJR Am J Roentgenol. 2000;174:523–7. 10658734 10.2214/ajr.174.2.1740523

[20] Long R, Wong E, Barrie J. Bronchial anthracofibrosis and tuberculosis: CT features before and after treatment. AJR Am J Roentgenol. 2005;184:S33–6. 15728014 10.2214/ajr.184.3_supplement.01840s33

[21] Andreu J, Cáceres J, Pallisa E, Martinez-Rodriguez M. Radiological manifestations of pulmonary tuberculosis. Eur J Radiol. 2004;51:139–49. 15246519 10.1016/j.ejrad.2004.03.009

[22] Wu CY, Hu HY, Pu CY, Huang N, Shen HC, Li CP, et al Aerodigestive tract, lung and haematological cancers are risk factors for tuberculosis: an 8-year population-based study. Int J Tuberc Lung Dis. 2011;15:125–30. 21276308

[23] Denholm R, Schüz J, Straif K, Stücker I, Jöckel KH, Brenner DR, et al Is previous respiratory disease a risk factor for lung cancer? Am J Respir Crit Care Med. 2014;190:549–59. 10.1164/rccm.201402-0338OC 25054566 PMC4214084

