# Supplementary material for: Exposure to Mycobacterium tuberculosis during Flexible Bronchoscopy in Patients with Unexpected Pulmonary Tuberculosis
Source: PLoS One. 2016 May 26;11(5):e0156385. doi: 10.1371/journal.pone.0156385 (PMC4882062; doi:10.1371/journal.pone.0156385)
Supplement: S1 Table — PTB, pulmonary tuberculosis. (DOCX) [file pone.0156385.s001.docx]

| CT findings | Definition |
| --- | --- |
| Pneumonia | Consolidation which appears as homogeneous increase in lung parenchymal attenuation with obscuration of the vessels and airway walls [1]. |
| Bronchiolitis | Well-defined small nodules and branching centrilobular nodules, or tree-in-bud pattern [2]. |
| Lung nodule | Rounded or irregular opacity, measuring up to 3cm in diameter [3]. |
| Lung mass | Thoracic opacity measuring greater than 3cm in diameter [3]. |
| Anthracofibrosis | Bronchial narrowing or obstruction and/or bronchial wall thickening [4]. |
| Bronchiectasis | The presence of the visibility of a bronchus in the outer one third of the lung or a diameter of the inner lumen greater than that of the accompanying pulmonary artery [5]. |
| Atelectasis | Increased attenuation of the affected lung associated with reduced lung volume [3]. |
| Interstitial lung disease | Typical imaging findings of reticular opacities, honeycombing, and ground-glass opacity predominantly in the peripheral and basal lung areas [6]. |
| Airway stenosis | The presence of focal or diffuse narrowing of the lumen as compared with its proximal and distal bronchi for vertically oriented bronchi, or with the adjacent same-order bronchus for horizontally or obliquely oriented bronchi [7] |
| Pleural effusion | Concave or convex shaped fluid density within the visceral and parietal pleural layers [8]. |
| Fibrocalficied parenchymal PTB | Pulmonary nodules with calcification and architectural distortion, suggesting sequelae of previous tuberculosis [9]. |

PTB, pulmonary tuberculosis.

References

[1] Leung AN, Miller RR, Müller NL. Parenchymal opacification in chronic infiltrative lung diseases: CT-pathologic correlation. Radiology. 1993;188:209–14.

[2] Koh WJ, Lee KS, Kwon OJ, Jeong YJ, Kwak SH, Kim TS. Bilateral bronchiectasis and bronchiolitis at thin-section CT: diagnostic implications in nontuberculous mycobacterial pulmonary infection. Radiology. 2005;235:282–8.

[3] Hansell DM, Bankier AA, MacMahon H, McLoud TC, Müller NL, Remy J. Glossary of Terms for Thoracic Imaging. Radiology. 2008;246:697–722.

[4] Kala J, Sahay S, Shah A. Bronchial anthracofibrosis and tuberculosis presenting as a middle lobe syndrome. Prim Care Respir J. 2008;17:51–5.

[5] Eom JS, Lee G, Lee HY, Oh JY, Woo SY, Jeon K, et al. The relationships between tracheal index and lung volume parameters in mild-to-moderate COPD. Eur J Radiol. 2013;82:e867–72.

[6] Misumi S, Lynch DA. Idiopathic pulmonary fibrosis/usual interstitial pneumonia: imaging diagnosis, spectrum of abnormalities, and temporal progression. Proc Am Thorac Soc. 2006;3:307–14.

[7] Kim HY, Im JG, Goo JM, Kim JY, Han SK, Lee JK, et al. Bronchial anthracofibrosis (inflammatory bronchial stenosis with anthracotic pigmentation): CT findings. AJR Am J Roentgenol. 2000;174:523–7.

[8] Abramowitz Y, Simanovsky N, Goldstein MS, Hiller N. AJR Am J Roentgenol. 2009;192:618–23.

[9] Jeong YJ, Lee KS. Pulmonary tuberculosis:up-to-date imaging and management. JAR AM J Roentgenol. 2008;191:834–44.
